# Antibodies against endogenous retroviruses

**DOI:** 10.1111/imr.13378

**Published:** 2024-08-17

**Authors:** Mihaela Chisca, Jean‐David Larouche, Qi Xing, George Kassiotis

**Affiliations:** ^1^ Retroviral Immunology Laboratory The Francis Crick Institute London UK; ^2^ Department of Infectious Disease, Faculty of Medicine Imperial College London London UK

**Keywords:** autoimmunity, cancer, endogenous retrovirus, envelope glycoprotein, humoral immunity, immunogenicity

## Abstract

The human genome harbors hundreds of thousands of integrations of ancient retroviruses, amassed over millions of years of evolution. To reduce further amplification in the genome, the host prevents transcription of these now endogenous retroviruses (ERVs) through epigenetic repression and, with evolutionary time, ERVs are incapacitated by accumulating mutations and deletions. However, several members of recently endogenized ERV groups still retain the capacity to produce viral RNA, retroviral proteins, and higher order structures, including virions. The retention of viral characteristics, combined with the reversible nature of epigenetic repression, particularly as seen in cancer, allow for immunologically unanticipated ERV expression, perceived by the adaptive immune system as a genuine retroviral infection, to which it has to respond. Accordingly, antibodies reactive with ERV antigens have been detected in diverse disorders and, occasionally, in healthy individuals. Although they are part of self, the retroviral legacy of ERV antigens, and association with and, possibly, causation of disease states may set them apart from typical self‐antigens. Consequently, the pathogenic or, indeed, host‐protective capacity of antibodies targeting ERV antigens is likely to be context‐dependent. Here, we review the immunogenicity of typical ERV proteins, with emphasis on the antibody response and its potential disease implications.

## 1 INTRODUCTION

Retroviruses are a group of highly successful RNA viruses that have been co‐evolving with the vertebrate hosts for hundreds of millions of years.^1^ They derive their name from the obligatory step in their replication cycle of reverse transcription of their RNA genome into a DNA copy, which they insert into the DNA of the host cell. This proviral DNA copy becomes part of the genetic code of the target cell, subsequently passed on to daughter cells through successive cell divisions.^2^ Retroviruses can therefore amplify their copies by the typical cell‐to‐cell transmission of progeny virions, or through clonal expansion of an infected cell without the need for virus transcription or progeny virion production.^2^ When the infected cell is a germ cell, the proviral DNA can become part of the genetic constitution of the host species, passed down successive generations in Mendelian fashion.

Waves of retroviral germline invasion and further amplification over the course of evolution has resulted in the accumulation of a staggering number of proviral copies in vertebrate genomes.^2^, ^3^, ^4^ Collectively referred to as endogenous retroviruses (ERVs), they belong to a group of retrotransposable elements characterized by the presence of long terminal repeats (LTRs) at either ends of the viral genome, which also includes mammalian apparent LTR retrotransposons (MaLRs). In humans, over 700,000 copies of ERVs and MaLRs are recognized, amounting to 8.83% of our genome.^5^ The vast majority are incomplete copies with mutations or deletions, most commonly through recombination between the two LTRs, that render them replication‐defective and also remove their ability to produce retroviral proteins.^2^, ^3^ Nevertheless, hundreds of ERV copies in the human genome have retained partial or complete open reading frames (ORFs) for canonical retroviral proteins, with the best conserved ORFs found in the most recently endogenized groups.^2^

In addition to genetic disintegration, epigenetic control prevents provirus expression, albeit imperfectly and reversibly.^6^ This reversibility and the retention of intact ORFs by numerous proviruses can lead to the expression of retroviral proteins, as well as higher order structures, such as virions, which can be immunogenic. Indeed, adaptive immune responses against diverse ERV proteins have been reported in autoimmune, neoplastic and neurodegenerative disorders.^7^ However, their precise targets, triggers and consequences are still incompletely understood. Here, we review the current evidence for the induction of and potential role for B cell responses to ERVs, particularly in the context of cancer.

## 2 ERV ANTIGENS TARGETED BY ANTIBODIES

**FIGURE 1:**
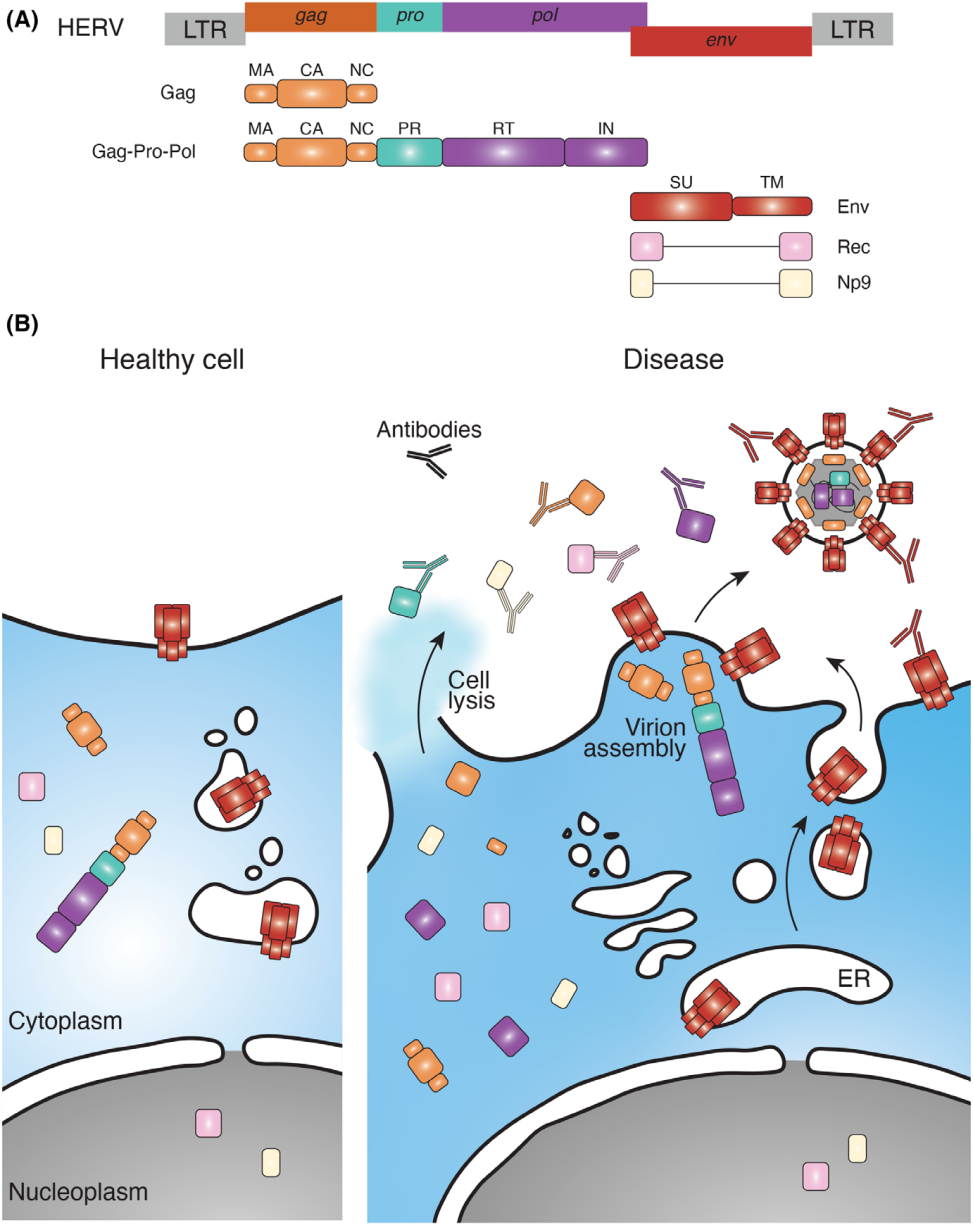
Canonical HERV‐derived antigens. (A) Genomic structure of a typical HERV provirus and arrangement of the *gag*, *pro*, *pol*, and *env* ORFs. Also shown are the Gag, Gag‐Pro‐Pol, and Env polypeptides produced from the translation of these ORFs, and the individual proteins that result from their proteolytic processing, as well as of Rec and Np9 made from alternatively spliced mRNA of HERV‐K (HML‐2) proviruses specifically. (B) Subcellular localization of canonical HERV proteins in a healthy or diseased/lysed cell and access to reactive antibodies. Antibodies are colored according to their target specificity.

By analogy to their exogenous precursors, ERVs in the human genome (HERVs) exhibit the typical retroviral genome structure, with two LTRs flanking the internal sequences containing the canonical *gag*‐*pro*‐*pol* and *env* ORFs.^2^ Translation of the unspliced HERV RNA transcript produces the group‐specific antigen (Gag), protease (Pro), and polymerase (Pol) proteins, which are further processed by the viral Pro (Figure 1). Proteolytic processing of Gag produces the structural proteins matrix (MA), capsid (CA), and nucleocapsid (NC), whereas processing of Pol produces the reverse transcriptase (RT) and integrase (IN) enzymes (Figure 1).^2^ The envelope glycoprotein (Env) is translated from a separate, spliced mRNA transcript and is also proteolytically processed, typically by the cellular Furin or Furin‐like proteases, to yield the surface unit (SU) and transmembrane unit (TM), which remain linked by disulfide bonds.

The most recently integrated proviruses, such as members of the HERV‐K mammary tumor virus (MMTV)‐like 2 (HML‐2) group, have retained the most intact ORFs for all typical retroviral proteins.^2^ These complex HERV‐K (HML‐2) retroviruses encode additional proteins, such as a spacer peptide (SP1) and glutamine‐ and proline‐rich peptides, (QP1 and QP2, respectively), resulting from the processing of Gag.^8^ Through secondary splicing of the *env* mRNA, certain HERV‐K (HML‐2) proviruses also encode Rec (Figure 1), a protein that mediates nuclear export of unspliced retroviral RNA, similarly to the function of Human immunodeficiency virus‐1 (HIV‐1) Rev.^9^ HERV‐K (HML‐2) proviruses, as well as those from most other HERV‐K groups, also encode dUTPase, a third enzyme that may contribute to the fidelity of reverse transcription.^10^ A 292‐bp deletion in the junction between the *pol* and *env* ORFs of certain HERV‐K (HML‐2) proviruses that are referred to as Type 1, truncates the Env protein, creates a novel protein termed Np9 from the mRNA that would otherwise produce Rec,^11^ and also removes the termination codon at the end of the *pol* ORF, thereby creating a Pol–Env fusion polypeptide.^12^

As with exogenous retroviruses, the production of canonical HERV proteins from translation of a polycistronic RNA and from processing of longer polypeptides determines their stoichiometry, and specific signals ensure their appropriate localization in the cell and virion. In turn, localization determines the degree to which HERV proteins are exposed to the adaptive immune system, and to antibodies in particular, with implications for their immunogenicity (Figure 1). For example, whereas Env glycoproteins are by necessity expressed at the plasma membrane of the cell or of the virion, the remaining HERV proteins are strictly in intracellular and intravirion compartments or only in intracellular compartments. Such proteins would only be exposed to antibodies following lysis of cells or virions that contain them.

It is important to note that the properties of canonical retroviral proteins may not apply to all HERV‐produced proteins, especially to non‐canonical products such as the Pol–Env fusion polypeptide of Type 1 HERV‐K (HML‐2) proviruses. Moreover, owing to accumulated mutations and deletions over the long periods of time since their integration, most HERVs have lost the ability to produce the canonical retroviral proteins and, consequently, to form virions. They may still produce mutated, truncated, or extended versions of canonical proteins. The altered stability, and intracellular trafficking and localization of such aberrant HERV proteins would affect their immunogenic potential. For instance, the N‐terminal truncation of Type 1 HERV‐K (HML‐2) Env proteins removes the putative signal peptide and mutations in most full‐length Type 2 HERV‐K (HML‐2) Env proteins affect processing, glycosylation and trafficking, resulting in increased retention in the endoplasmic reticulum and reduced export to the plasma membrane.^13^, ^14^, ^15^ The relatively well‐documented example of Types 1 and 2 HERV‐K (HML‐2) Env proteins will also apply to many more aberrant versions of canonical retroviral proteins, which represent the majority of those produced by HERVs, and which have not yet been studied in the same degree of detail. By extension, the ability of HERV proviruses to form virions, either individually or collectively by complementation,^16^ composed of or incorporating noncanonical HERV proteins is still incompletely studied. Therefore, it is often difficult to discern which particular copy or variant of a HERV protein may have triggered a B‐cell response and to which extent such B‐cell responses may cross‐react with other copies or variants of the same protein.

Typically, B‐cell responses to HERV proteins are detected by assays using a single representative protein, or peptides thereof, which may variably capture the breadth of antibody responses to protein variants. Nevertheless, antibody response to several individual HERV proteins have been repeatedly detected in diverse settings and by a variety of methods (Table 1). Detection methods include Western blotting (WB), enzyme‐linked immunosorbent assay (ELISA), Luminex, and time‐resolved immunofluorometric assay (TR‐IFMA) using recombinant HERV proteins, domains or synthetic peptides as targets, or immunofluorescence (IF) and flow cytometry (FC)‐based assays using intact HERV proteins expressed in target cells (Table 1). Antibody targets include both cell surface and intracellular HERV proteins and although antibody responses to HERV‐K (HML‐2) Gag, Env, Rec, and Np9 in cancer patients are the most frequently reported, antibodies to Env and Gag proteins from other HERVs, including HERV‐E, HERV‐H, HERV‐W and Human T‐lymphotropic virus (HTLV)‐related endogenous sequence‐1 (HRES‐1) have also been detected (Table 1).

**TABLE 1:** Studies reporting the detection of HERV‐reactive antibodies.

Target(s)	Detection	Cohort	Study
HERV‐K (HML‐2) Env	WB	BRCA, HIV‐1 infection, CMV infection	Vogetseder et al.^17^
HERV‐K (HML‐2) Gag, Rec, Env	ELISA	TGCT, AML, CML, pregnant women, HIV‐1 infection	Denner et al.^18^
HERV‐K (HML‐2) Gag	IF	TGCT	Sauter et al.^19^
HERV‐K (HML‐2) Gag	WB	TGCT, pregnant women	Boller et al.^20^
HERV‐E Gag	WB	SLE, SjS, MCTD	Hishikawa et al.^21^
HERV‐K (HML‐2) Env	ELISA	SLE, SjS	Herve et al.^22^
HERV‐H Gag, Env	TR‐IFMA	MS	Christensen et al.^23^
HERV‐K (HML‐2) Gag, Env	IF, WB	TGCT	Kleiman et al.^24^
HERV‐K (HML‐2) Env	WB	SKCM	Buscher et al.^25^
HERV‐K (HML‐2) Env, Gag	ELISA, IF	SKCM	Humer et al.^26^
HERV‐K (HML‐2) Env HERV‐E Env, ERV3‐1 Env	ELISA	OV	Wang‐Johanning et al.^27^
HERV‐K (HML‐2) Gag, Env	IF, WB	SKCM	Hahn et al.^28^
HERV‐H Env HERV‐W Env	TR‐IFMA	MS	Brudek et al.^29^
HERV‐K (HML‐2) Pol	ELISA	LGLL	Thomas et al.^30^
HERV‐K (HML‐2) Gag	ELISA	PRAD, SKCM, BRCA, OV	Reis et al.^31^
HERV‐K (HML‐2) Gag, Pol	ELISA	HTLV myelopathy, LGLL, MS	Perzova et al.^32^
HERV‐K (HML‐2) Env, Rec, Np9	ELISA	BRCA, ductal carcinoma in situ	Wang‐Johanning et al.^33^
HERV‐K (HML‐2) Env, Gag	ELISA	Psoriasis	Gupta et al.^34^
HERV‐K (HML‐2) Gag	ELISA	RA	Nelson et al.^35^
HERV‐K (HML‐2) Env	ELISA	HIV‐1 infection	Michaud et al.^34^
HRES‐1 Gag	ELISA	HTLV myelopathy, HTLV‐1 or ‐2 infection	Perzova et al.^36^
HERV‐K (HML‐2) Env	ELISA	RA	Mameli et al.^37^
HERV‐K (HML‐2) Gag	ELISA	HIV‐1 infection	de Mulder et al.^38^
HERV‐K (HML‐2) Env HERV‐W Env	ELISA	ALS, MS, AD	Arru et al.^39^
HERV‐K (HML‐2) Env	ELISA, Luminex	SLE	Tokuyama et al.^40^
HERV‐K (HML‐2) Env ERV3‐1 Env	FC	JSLE, JIA, JDM, MIS‐C	Deakin et al.^41^
HERV‐K (HML‐2) Env	ELISA	ALS	Simula et al.^42^
HERV‐K (HML‐2) Env	ELISA	ALS	Arru et al.^43^
HERV‐K (HML‐2) Env	ELISA	ALS	Garcia‐Montojo et al.^44^
HERV‐K (HML‐2) Env	ELISA	SLE, JSLE	Khadjinova et al.^45^
HERV‐K (HML‐2) Env	WB, ELISA	RA	Wang et al.^46^
HERV‐W Syncytin‐1	ELISA	SLE	Lu‐Culligan et al.^47^
HERV‐K (HML‐2) Env	ELISA	T1DM	Noli et al.^48^
HERV‐K (HML‐2) Env HERV‐W Env, HERV‐H Env	ELISA	PRAD, benign prostate hyperplasia	Manca et al.^49^
HERV‐K (HML‐2) Env	FC	LUAD	Ng et al.^50^

Abbreviations: AML, acute myeloid leukemia; BRCA, breast invasive carcinoma; CML, chronic myeloid leukemia; LGLL, large granular lymphocytic leukemia; LUAD, lung adenocarcinoma; OV, ovarian serous cystadenocarcinoma; PRAD, prostate adenocarcinoma; SKCM, skin cutaneous melanoma; TGCT, testicular germ cell tumors.

Extensive HERV immunogenicity is also suggested by the diversity of conditions in which HERV‐targeting autoantibodies are detected. In addition to multiple types of cancer where they are most prevalent, HERV‐reactive antibodies have also been reported is several autoimmune and degenerative disorders, which include adult and juvenile systemic lupus erythematosus (SLE and JSLE, respectively), rheumatoid arthritis (RA) and juvenile idiopathic arthritis (JIA), juvenile dermatomyositis (JDM), Sjögren's syndrome (SjS), mixed connective tissue disease (MCTD), Type 1 diabetes mellitus (T1DM), multiple sclerosis (MS), amyotrophic lateral sclerosis (ALS, also known as motor neuron disease), and Alzheimer's disease (AD) (Table 1). They have also been found in patients infected with HIV‐1, HTLV‐1 or ‐2, or cytomegalovirus (CMV), and in pediatric patients who develop multisystem inflammatory syndrome in children (MIS‐C) following infection with severe acute respiratory syndrome coronavirus 2 (SARS‐CoV‐2) (Table 1). Together, these reports underscore the notion that immunogenicity may be a general feature of HERV antigens, applicable to multiple groups, rather than being an exceptional property of a single group.

## 3 TOLERANCE AND IMMUNOGENICITY OF ERVS

The frequent detection of antibodies to a multitude of HERV antigens highlights their natural immunogenicity in a range of conditions. These observations raise questions with respect to the degree of immunological tolerance that HERV antigens may induce or whether they are subject to the same immunological rules that apply to other self‐proteins.

At the transcriptional level, HERVs appear to be transcribed, constitutively and inducibly, in many healthy tissues, including the thymus,^51^, ^52^, ^53^, ^54^ and HERV transcripts are read by ribosomes,^55^ which suggests that a certain level of central tolerance would be induced. However, provirus transcription may not necessarily signify protein production, as there are several layers of control between the two. For example, similarly to their exogenous counterparts, provirus transcription of HERVs generates several alternatively or partially spliced RNA species, corresponding to genomic RNA or mRNAs that would be translated to distinct protein products. Moreover, nuclear export of partially spliced mRNA,^9^ rate of translation, as well as stability and trafficking of the resultant proteins^13^, ^14^, ^15^ also determine the correlation between RNA and protein abundance.

Whereas, RNA abundance can be measured reasonably well, the relative paucity of reagents for the detection of HERV protein products has so far limited accurate estimation of their abundance in healthy or diseased tissues. Nevertheless, HERV‐K (HML‐2) Env has been detected in the human thymus,^52^ where it may contribute to central tolerance. Studies from mice have demonstrated that *Emv2*, the single‐copy endogenous ecotropic MLV in the commonly used C57BL/6 strain, mediates at least partial central tolerance,^50^, ^56^ but does not prevent the induction of strong and protective antitumor responses against the *Emv2* Env and its recombinants that are frequently seen in mouse cancer models.^50^, ^57^ Evidence for thymic selection mediated by ERVs is also provided by superantigen activity exhibited by certain ERV Env glycoproteins.^58^ For example, endogenous MMTV proviruses encode for a superantigen that induces clonal deletion of TCR Vβ5^+^ thymocytes,^59^ while it spares developing regulatory T (Treg) cells, which, therefore, become enriched in ERV superantigen‐reactive clones.^60^, ^61^, ^62^ Superantigen activity has also been reported for a HERV‐K (HML‐2) provirus integrated into the human *CD48* gene that may induce deletion of TCR Vβ7^+^ thymocytes.^63^, ^64^, ^65^, ^66^ However, the degree to which the human TCR repertoire is shaped by thymic expression of this particular provirus remains unclear.

Although formation of the BCR repertoire is also subject to deletional tolerance, some autoreactive B cell clones may persist in the repertoire, remaining tolerized by non‐deletional mechanisms, including the lack of T cell help.^67^, ^68^ Under such circumstances, breaking of T helper cell tolerance to ERV antigens is expected to lead to breaking also of B‐cell tolerance to the same antigens. This phenomenon is exemplified in studies of vertical transmission of infectious recombinants of endogenous MLVs, where pups born to infected immunodeficient dams experience multiple levels of tolerance.^69^ Their TCR and BCR repertoires are subject to central tolerance through expression of the germline copy of *Emv2* Env, as well as to neonatal tolerance through transmission of fully infectious MLVs at or shortly after birth, which then cause systemic infection.^69^ Despite these two severe forms of tolerance, such neonatally infected and centrally tolerized mice still mount an antibody response to *Emv2* Env. Notably, progeny of such mice, which inherit infectious MLVs, as well as maternal antibodies, mount an even stronger antibody response to the infecting virus, and the amplification of these immune responses over three to four successive mouse generations leads to the purging of the infectious virus, breaking both tolerance and the chain of transmission.^69^ These experiments demonstrated that antibody responses to ERVs, even when fully infectious, can be induced in the face of extreme immunological tolerance and can also be significantly accelerated by the availability of help from non‐tolerized T helper cells.^69^

Endogenous MLV‐targeting autoreactive B‐cell responses have long been observed in mice^70^ and are necessary to prevent the generation and subsequent spread of infectious MLV recombinants.^71^, ^72^, ^73^ The ability of endogenous MLVs to break immunological tolerance has recently been linked to their ability to display their antigens on the surface of producer cells and of virions.^73^ Indeed, the integration into the mouse genome of a replication‐competent MLV provirus, in which a GFP reporter protein was inserted into the proline‐rich region of the Env ectodomain, leads to the spontaneous induction of anti‐GFP antibodies.^73^ In contrast, expression of GFP as a separate protein by a similar provirus does not induce anti‐GFP antibodies, suggesting that the immunogenicity of GFP is determined by its inclusion as a part of ERV proteins displayed on the surface of cells and virions.^73^ These findings from the study of endogenous MLVs may explain the level of immunogenicity reported for HERVs, where few members encode for proteins that can be displayed on the cell surface and even fewer can form virions. They may also explain the induction of HERV antibodies following infection with HIV‐1 or HTLV‐1, where HERVs can be transcriptionally transactivated thought the action of HIV‐1 Tat and HTLV‐1 Tax,^74^, ^75^, ^76^ but may also participate in the formation of virions produced by the infectious retroviruses.^77^, ^78^

Infection with viruses other than retroviruses may also transactivate HERV expression,^79^, ^80^ compromising tolerance of HERV antigens. In addition to direct transactivation, excessive inflammation caused by viral infections may also contribute to the breaking of immunological tolerance to HERV antigens. For example, the development of MIS‐C in children following SARS‐CoV‐2 infection, which leads to production of HERV‐K (HML‐2) Env‐reactive antibodies,^41^ is linked to deficiencies in OAS1, OAS2, or RNase L that lead to overproduction of inflammatory cytokines.^81^ Tolerance to HERV antigens is also compromised in the setting of systemic autoimmunity, as it may be expected for self‐antigens more generally. However, the inducible nature of HERV transcription through responsiveness to inflammation and epigenetic changes in infection or cancer, combined with the retention of viral characteristics, such as the export and display of Env glycoproteins and formation of virions may promote their immunogenicity and set them apart from other classes of self‐antigen.

## 4 DISEASE IMPLICATIONS OF ERV ANTIBODIES

Irrespective of the mechanisms by which immunological tolerance of HERV antigens may be overcome, antibodies to this class of non‐mutated self‐antigen is by definition autoimmune in nature. As such, the implications for the disorders, in which HERV‐reactive antibodies are found is not immediately obvious and can range from beneficial to detrimental. The possible effect of HERV‐reactive antibodies also heavily depends on the pattern of expression and putative function of their specific HERV antigen target. Neither of these properties of HERVs are well understood, but parallels may be drawn with exogenous retroviruses.

Retroviral infection is most commonly associated with the development of malignancies.^82^ Infectious retroviruses in humans and in other species can cause cancer by multiple mechanisms, including insertional mutagenesis, induction of immunodeficiency, transduction of oncogenes they may carry, or by the transforming nature of their own proteins, such as the Env glycoprotein of Jaagsiekte sheep retrovirus (JSRV), causing lung cancer.^83^, ^84^ Although rarely providing sterilizing immunity, the adaptive immune response to retroviral infection can limit the level of infection and, therefore, provide a certain degree of protection from ensuing cancer. Moreover, infected and transformed cells may continue to express retroviral antigens, even if they lose the ability to produce infectious virions, and such retroviral antigens can serve as tumor‐specific antigens, again turning the antiviral response into an antitumoral response.

One example, documented several decades ago, is the FBL‐3 murine leukemia transplantable cell line, originally induced by infection of mice with Friend virus (FV), a retroviral complex of Friend helper MLV (F‐MLV) and spleen focus‐forming virus (SFFV). Although FBL‐3 cells do not produce infectious virions, they do carry and express F‐MLV and SFFV proviruses and this expression generates tumor antigens of retroviral origin, in turn triggering highly protective antitumor T‐cell and antibody responses against the F‐MLV Env glycoprotein.^85^, ^86^, ^87^, ^88^

This same principle also operates at the level of germline proviruses that are transcriptionally reactivated in cancer cells, given the incomplete induction of immunological tolerance to their antigens. The targeting of tumor antigens, generated by the expression of ERV proteins in cancer cells has also been documented several decades ago.^89^, ^90^ Here, expression of Env glycoproteins from endogenous MLVs in B16 melanoma cells has been shown to induce strong Env‐specific antibody responses that restrain growth and metastasis of B16 melanomas.^89^, ^90^ However, despite originating from replication‐defective endogenous MLVs, the retrovirus responsible for the expression of the antigens in B16 melanomas that are targeted by the antitumor antibody response is fully infectious.^90^, ^91^ Such infectious MLVs arise through recombination of defective endogenous precursors and have been detected in numerous murine cell lines.^50^, ^57^, ^90^

By comparison with expression of defective endogenous MLVs alone, the presence of infectious MLVs in transplantable murine cancer cell lines likely increases their immunogenicity, through a substantial increase of the level of retroviral protein expression in cancer cells,^57^ and through the formation of retroviral particles, which are inherently more immunogenic. It will, therefore, be important to evaluate the immunogenicity of retroviral proteins and protective capacity of the antibody response that targets them, when these are expressed from defective ERVs, and not from fully infectious retroviruses that may confound murine cancer models.^57^ The absence of infectious retroviruses would also render murine cancer models more representative of human cancer, where HERV infectivity appears to have long been lost.

Despite their inability to complete the replication life cycle, transcriptional induction of HERV‐K (HML‐2) proviruses can lead to the formation of retroviral particles in human healthy tissues such as the placenta,^92^, ^93^, ^94^ cancer cell lines, such as teratocarcinomas^95^ and melanomas,^96^ and in the circulation of patients with lymphomas.^97^, ^98^ Formation of retroviral particles, would enhance the immunogenicity of this particular HERV group, by comparison with expression of individual proteins in cancer cells. However, it remains to be established whether the potentially increased immunogenicity of human proviruses that can form defective retroviral particles can match that of murine proviruses that can from fully infectious particles or whether it is sufficient to provide a target for an effective antitumor immune response.

A protective effect of antibodies against HERV‐encoded antigens expressed in cancer cells is supported by animal models. For example, a monoclonal antibody against HERV‐K (HML‐2) Env glycoproteins has been reported to induce apoptosis and to inhibit the growth of human breast cancer cell lines xenotransplanted into immunodeficient mice.^99^ Whether or not HERV‐reactive antibodies may play similar roles in human cancer is still uncertain, however, in part due to the scarcity of clinical data. In order to provide effective protection against tumors, HERV‐reactive antibodies would have to be induced sufficiently early during tumor development, and target antigens that are displayed on the surface of cancer cells also early during tumor progression and that are not subsequently lost due to tumor immune escape or other processes.

Antibodies to intracellular antigens that are released following lysis of tumor cells may contribute to tumor control in vivo,^100^ and it is, therefore, possible that antibodies to intracellular or intravirion HERV proteins, such as Gag, Rec, and Np9, also exert some antitumor activity. However, strictly intracellular antigens cannot provoke certain effector functions of antibodies, such as antibody‐dependent cellular cytotoxicity (ADCC), reducing their full antitumor potential. Instead, antibodies to intracellular HERV antigens may follow the extent of tumor growth and spread. Consistent with the latter idea, antibodies to HERV‐K (HML‐2) Gag were found more frequently in prostate cancer patients with more advanced than with early disease, and their presence was correlated with worse prognosis.^31^ Similar findings were reported for antibodies to HERV‐K (HML‐2) Env in patients with testicular germ cell tumors and seminomas, where the immune privilege of the testis may compromise any antitumor immune effects. In this indication, antibodies to HERV‐K (HML‐2) Env declined within a year after resection of the primary tumor or chemotherapy, and their retention after chemotherapy was associated with worse prognosis and the presence of residual primary tumor cells or metastases.^20^, ^24^

Antibodies to HERV‐K (HML‐2) antigens were found at higher levels in more advanced stages of melanoma, where they were associated with reduced survival, even after confirmed seropositivity.^28^ However, this analysis combined seroreactivity either to Gag or to Env, potentially masking a differential effect of the two specificities. Indeed, only a minority of melanoma patients had antibodies both to Gag and to Env,^28^ suggesting distinct processes leading to their induction. A potential disconnect between Gag and Env targeting antibodies may also explain the comparable positivity for HERV‐K (HML‐2) Env antibodies in early and late stage melanoma reported in other studies^26^ Antibodies to HERV‐K (HML‐2) Env SU, Rec, and Np9 have also been detected in ductal carcinoma in situ, the earliest stage of breast cancer, and were increased in later stage invasive ductal carcinoma.^33^

Their limitations notwithstanding, these studies collectively suggest that much of the antibody response to HERV antigens, particularly intracellular ones, exhibits a neutral effect on tumors, with antibody levels simply reflecting, rather than affecting, tumor burden. Alternatively, antibodies to HERV antigens may reach potentially protective levels late during tumor progression, thereby missing the opportunity to restrain tumor growth. It is also possible that at least some HERV antigen‐reactive antibodies have pro‐tumoral effects. The Env glycoproteins of HERV‐K (HML‐2) and HERV‐H have been shown to initiate signaling events that promote tumor growth and metastasis.^101^, ^102^, ^103^, ^104^, ^105^, ^106^ Studies with MLV have also indicated that ligation of retroviral Env proteins by antibodies may potentiate such signaling activities,^107^ thereby amplifying a pro‐tumoral effect.

Antibody responses to HERV antigens may be slow or ineffective in preventing cancer initiation of progression. They may nevertheless contribute to anticancer immunity, particularly in the context of cancer immunotherapies, which unleash the full potential of adaptive immunity and may also trigger autoimmune reactions. Consistent with this notion, higher mRNA transcription levels of HERV‐K (HML‐2) proviruses prior to immunotherapy have been reported to predict a favorable outcome in pancreatic and colorectal cancers^108^ and in lung adenocarcinoma,^50^ although the precise underlying mechanism and contribution of HERV‐reactive antibodies to this association remain unclear.

Although elucidation of protective, pathogenic or neutral effects of HERV‐reactive antibodies will be aided by further studies, their autoimmune nature suggests that some clues may be provided by comparisons with the effect of autoantibodies to other self‐antigens. While autoimmune reactions are considered, in principle, to be pathogenic, they may contribute to immune protection against cancer, where autoantigens are frequently overexpressed as non‐mutated tumor‐associated antigens.^109^

## 5 CANCER AND B‐CELL AUTOIMMUNITY

The relationship between antibody autoimmunity and cancer is intricate, as both conditions are closely related to immune function. The dysregulation of the immune response in autoimmunity may create a favorable environment for cancer cells to thrive. A plethora of studies have shown that patients with B cell‐mediated autoimmune diseases, such as RA, SLE, SjS, or Hashimoto's thyroiditis are more prone to developing malignancies.^110^, ^111^, ^112^, ^113^, ^114^, ^115^ Two non‐mutually exclusive mechanisms have been proposed to explain how heightened and prolonged immune activation in autoimmunity may promote cancer. First, B cells exhibiting clonal similarity to autoantibodies and extensive intraclonal diversity were identified in lymphoma patients with a prior history of Hashimoto thyroiditis and SjS, respectively,^116^, ^117^ suggesting that B cells underwent somatic hypermutation following antigenic stimulation. Somatic hypermutation can cause B‐cell transformation by aberrantly targeting oncogenes and causing genomic instability.^118^, ^119^ This notion is also consistent with the observation that B‐cell lymphomas are more frequent in patients with B cell‐mediated autoimmunity.^120^, ^121^

Another probable mediator of cancer in autoimmunity is chronic inflammation, a phenomenon closely linked to autoimmune disease and a well‐documented risk factor for cancer.^122^, ^123^ Indeed, it has been observed that patients with tissue‐specific immune‐mediated diseases frequently develop cancers locally, whereas systemic diseases had positive associations with cancers in distant organs.^124^, ^125^, ^126^ Notably, a lack of immune response may also promote cancer development.^127^ Therefore, the administration of immunosuppressive drugs, while alleviating symptoms of autoimmune disease, favors immune evasion.^128^, ^129^

In addition to a predisposing effect of autoimmunity to development of certain malignancies, it was observed that, conversely, cancers can elicit immune disorders named paraneoplastic neurological syndromes (PNS), which are autoimmune in nature. Overall, <1% of cancer patients develop PNS,^130^, ^131^ although the incidence can go up to 9% in small cell lung cancer (SCLC).^132^ These disorders occur when neuronal autoantigens expressed by tumor cells cause autoimmunity targeting the nervous system. Several autoantibodies identified in PNS patient sera recognize intracellular neuronal antigens, such as the breast and ovarian cancers antigen CDR2,^133^, ^134^ the small cell lung cancer antigens HuC and HuD,^135^ or the paraneoplastic Ma antigens (PNMA) Ma1 and Ma2.^136^, ^137^ Interestingly, the PNMA group of genes, many of which exhibit neuron‐ and testis‐specific expression, are derived from the *gag* genes of Ty3/Gypsy LTR retrotransposons that have been domesticated multiple times during evolution and co‐opted as regulators of apoptosis.^138^, ^139^ The retroviral origin of the PNMA group of antigens raises the possibility that HERV antigens, including PNMA, overexpressed in tumor cells trigger antibody responses that cross‐react with antigens in similar proteins co‐opted in physiological functions, thereby contributing to PNS. It should be noted, however, that the mere expression of autoantigens by tumor cells may be insufficient to induce PNS, as tumors frequently express autoantigens, including autoantigens of retroviral origin, but autoimmunity and PNS only arise in a minority of cases. For instance, >85% of SCLC patients fail to develop spontaneous antitumor immunity even though their tumors express the HuC and HuD antigens.^140^ Further investigation will be required to identify the precise factors needed to overcome immunological tolerance and cause spontaneous autoimmunity in cancer. Interestingly, it was observed that cancer immunotherapies, and more specifically immune checkpoint blockade (ICB), stimulate antitumor immunity by circumventing the tolerance mechanisms of immune cells and can, as such, cause breaches in B‐cell and T‐cell tolerance. Immune checkpoint molecules promote T‐cell exhaustion, and regulate B‐cell activation^141^ and clonal selection during somatic hypermutation and affinity maturation.^142^, ^143^ ICB has been shown to cause immune‐related adverse effects (irAE)—heterogeneous toxicities ranging from fatigue, rash, and nausea to potentially fatal conditions such as hepatitis and myocarditis—in around 50% of melanoma patients 1 year after treatment.^144^ Hence, both autoimmunity and cancer frequently co‐occur in patients as the inherent properties of these pathologies and the immunomodulating nature of their treatment disrupt the regulation of the immune response.

Whether concomitant antibody autoimmunity has a beneficial or pathological impact on cancer prognosis is a long‐standing conundrum. Research on how autoimmunity affects cancer survival is complex, as several studies must combine either autoimmune disorders or cancer types due to low comorbidity frequency. Additionally, preexisting and cancer‐ or treatment‐induced autoimmunity—such as PNS and irAE—must be distinguished as their etiology differs greatly. Preexisting autoimmune diseases are associated with worse prognosis for acute lymphoblastic, chronic lymphocytic, acute myeloid, and chronic myeloid leukemias.^145^ Autoimmunity is also linked to higher mortality in digestive tract cancers, except for RA, which improves survival in colon and rectal cancers.^146^ Another study reported a better prognosis for small bowel cancer patients with celiac disease.^125^ Diagnosis of 10 distinct autoimmune diseases improved the overall survival of Stages I, III, and IV SCLC and non‐small cell lung cancer (NSCLC) patients, suggesting autoimmunity could have a beneficial prognosis effect at all stages of malignancy.^147^ A similar observation in breast cancer supports this, where multivariable regression analysis revealed increased overall survival and decreased cancer‐specific mortality in patients with autoimmunity.^148^ Thus, even though it has been abundantly reported that pre‐existing autoimmunity promotes cancer formation, these studies show that autoimmune diseases generally improve cancer prognosis. However, this could depend highly on the mechanism of the autoimmune disease. The analysis of an international cohort of lymphomas revealed that B cell‐mediated autoimmune disorders increased the survival of Hodgkin and mantle cell lymphoma patients, whereas T cell autoimmunity did not significantly impact the survival of any lymphoma subtype.^120^ This suggests that autoantibodies might be central to the protective effect of autoimmunity in cancer. The analysis of additional large cohorts of patients is deeply required to dissect the impact of individual autoimmune diseases on cancer prognosis.

On the other hand, PNS and irAE are cancer‐ and treatment‐induced autoimmune disorders, respectively. Even though they do not initiate the antitumor response, they are the symptom of a robust immune activation against malignant cells. PNS, which target the nervous system, are typically associated with poorer prognosis as they can cause long‐term or permanent motor and cognitive symptoms or death.^131^, ^149^, ^150^ An exception is the Lambert‐Eaton myasthenic syndrome, which affects the neuromuscular junctions and is associated with better survival in SCLC patients.^151^ This is consistent with the “beneficial autoimmunity” hypothesis, which states that autoimmunity is deleterious when targeting essential cells and beneficial when targeting nonessential ones.^152^ Accordingly, although irAE are generally associated with improved response to ICI and overall survival in metastatic NSCLC, head and neck cancer, and melanoma,^153^, ^154^, ^155^ it has been reported that pulmonary irAE were associated with worse response to ICI, whereas skin, gastrointestinal, and endocrine irAE improved overall response.^156^

A beneficial outcome of ICB therapies is increasingly associated with the formation of tertiary lymphoid structures (TLS), which are ectopically formed lymphoid organs, in and around solid tumors.^157^, ^158^ TLS may promote the production of antitumor antibodies, including antibodies targeting ERV antigens,^50^ but also support the local induction of antitumor cytotoxic T‐cell responses.^157^, ^158^ TLS are a frequent feature of autoimmune disease and other disorders of chronic inflammation, particularly of the targeted organs, where they support the induction of local immune responses.^159^, ^160^ It is, therefore, possible, that preexisting TLS or those associated with autoimmunity or chronic inflammation may also facilitate the induction of local antitumor responses in the affected organs. Whether the “beneficial autoimmunity” principle can also be applied to preexisting autoimmune diseases remains to be assessed and will provide new insights on cancer treatment and outcome.

## 6 CONCLUSIONS AND FUTURE DIRECTIONS

**FIGURE 2:**
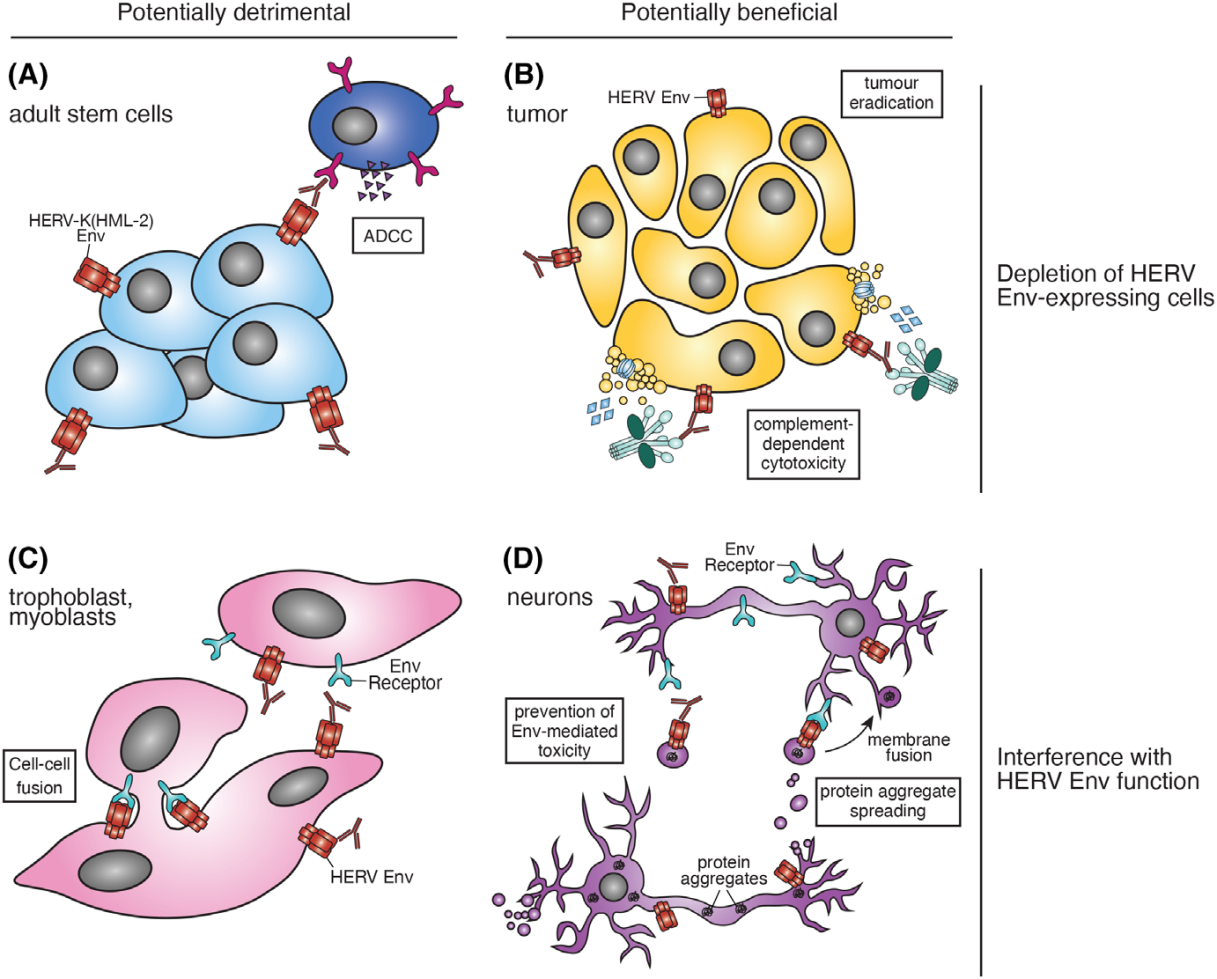
Possible effects of HERV Env‐reactive antibodies. (A, B) Antibodies binding to cell surface‐expressed HERV Env may lead to the elimination, thought ADCC or complement fixation of healthy (e.g., adult stem cells) or diseased cells (e.g., tumor cells), with potentially detrimental or beneficial consequences. (C, D) Antibodies blocking the attachment of HERV Env to its receptor(s) on target cells may interfere with physiological cell–cell fusion (e.g., of trophoblasts or myoblasts) or intercellular transfer of pathogenic cargo (e.g., seeds of protein aggregation or signals of senescence). They may also block potentially toxic activities of HERV Env in expressing or on neighboring cells.

As with antibody responses to other classes of autoantigens,^152^ the balance of potentially pathogenic and protective effects of antibody responses to HERV antigens in cancer will depend on the essentiality of the cell types that express them (Figure 2). Another important distinction, however, is the function of the target protein itself, rather than of the cell that expresses it. Cellular proteins invariably play a physiological role, although some may be important only early in development or in restricted, sex‐specific tissues, such as the ovaries or testes. Essential physiological functions can also be carried out by HERV‐derived proteins, such as Syncytins, which are captured HERV Env proteins exapted in placentation^161^ and myogenesis.^162^ It has been argued that antibody responses to HERV Env proteins with an important function, such as Syncytins in trophoblasts, or expressed in other essential cells, such as the HERV‐K (HML‐2) Env in adult stem cells or thymic epithelial cells, may compromise pregnancy, tissue regeneration, or thymic selection (Figure 2).^163^ Although the evidence is currently lacking, it will be important to establish whether adult stem cells or other essential adult or embryonic cell types express sufficient levels of HERV‐encoded antigens to be eliminated or adversely affected by an anti‐HERV autoantibody response. Unfounded concerns relating to potential cross‐reactivity of antibodies raised by COVID‐19 vaccines against the SARS‐CoV‐2 spike glycoprotein and Syncytin‐1, which could, in theory, interfere with pregnancy, prompted thorough investigation of the levels of Syncytin‐1 autoreactivity in vaccinated and unvaccinated individuals. In contrast to reactivity to other HERV Env glycoproteins, very low prevalence of antibody reactivity to Syncytin‐1 has been identified in multiple studies,^47^, ^164^, ^165^ suggesting an inherent lack of overt immunogenicity of Syncytin‐1. It will be equally important to ascertain the precise antigenic specificity of HERV‐reactive autoantibodies seen in autoimmunity, cancer or other conditions, as such antibodies may target distinct HERV proteins with nonoverlapping patterns of expression. The sequence diversity among individual copies of a given HERV protein and potential cross‐reactivity between the copy that triggers the antibody response and all other related copies will also affect the pathogenic potential of such a response.

In contrast to cellular proteins and the exapted Syncytins, no physiological role for the majority of HERV‐encoded proteins has yet been described, arguing against the possibility that an autoimmune response targeting HERV antigens would endanger physiological functions. Moreover, certain HERV Env proteins may even exert pathogenic functions, such as the HERV‐K (HML‐2) Env in amyotrophic lateral sclerosis^166^ and in aging‐related inflammation,^167^ HERK‐W Env‐derived Syncytin 1 in multiple sclerosis,^168^ and HERV‐K (HML‐2) Env and Syncytin 1 in neurodegenerative diseases.^169^ Consequently, antibodies against these HERV Env proteins could potentially block their pathogenic function in these particular settings and provide a degree of protection from disease progression (Figure 2).^44^

The available evidence suggests that HERV‐reactive antibodies are induced in cancer, as well as in systemic autoimmunity, in infection with HIV‐1 or HTLV‐1, in MIS‐C developing after SARS‐CoV‐2 infection, and in neurodegenerative and neuroinflammatory disorders. Potentially pathogenic HERV‐targeting autoantibodies may also contribute to the toxicities of cancer immunotherapies. Aging, which is a strong cancer risk factor, is also associated with HERV derepression and elevated expression of HERV‐K (HML‐2) proviruses. Whether aging is also linked to increasing titers of HERV‐K (HML‐2)‐reactive antibodies will be interesting to explore. Shared targets of HERV‐reactive antibodies induced in these diverse disorders or naturally during aging may affect the interaction between them, with antibodies induced by one disease or condition affecting susceptibility or protection from another. Although larger studies will be required to discern such a possible effect, elucidating potential disease interactions through the natural induction of cross‐reactive HERV‐targeting antibodies will also be highly informative for any strategies aiming to administer such antibodies in a therapeutic setting or induce them through vaccination.

## AUTHOR CONTRIBUTIONS

GK conceived the article. MC, J‐DL, QX, and GK wrote the manuscript. MC, J‐DL, and GK prepared display items. All authors approve the submitted version of the article.

## CONFLICT OF INTEREST STATEMENT

GK is a scientific cofounder of EnaraBio and a member of its scientific advisory board. GK has consulted for EnaraBio and Repertoire Immune Medicines. The other authors declare no competing interests.

## Data Availability

Not applicable.
